# Dominance loss and tenure maintenance in Kalahari meerkats

**DOI:** 10.1093/beheco/arad066

**Published:** 2023-08-22

**Authors:** Chris Duncan, Jack Thorley, Marta B Manser, Tim Clutton-Brock

**Affiliations:** Large Animal Research Group, Department of Zoology, University of Cambridge, Downing Street, Cambridge CB2 3EJ, UK; Kalahari Research Centre, Kuruman River Reserve, Northern Cape 8467, South Africa; Large Animal Research Group, Department of Zoology, University of Cambridge, Downing Street, Cambridge CB2 3EJ, UK; Kalahari Research Centre, Kuruman River Reserve, Northern Cape 8467, South Africa; Kalahari Research Centre, Kuruman River Reserve, Northern Cape 8467, South Africa; Department of Evolutionary Biology and Environmental Studies, University of Zurich, Winterthurerstrasse 190, 8057 Zurich, Switzerland; Mammal Research Institute, University of Pretoria, 0028 Pretoria, South Africa; Large Animal Research Group, Department of Zoology, University of Cambridge, Downing Street, Cambridge CB2 3EJ, UK; Kalahari Research Centre, Kuruman River Reserve, Northern Cape 8467, South Africa; Mammal Research Institute, University of Pretoria, 0028 Pretoria, South Africa

**Keywords:** breeding lifespan, cooperative breeders, dispersal, dominance tenure, intrasexual competition, *Suricata suricatta*

## Abstract

In many social species, both the acquisition of dominance and the duration that individuals maintain their status are important determinants of breeding tenure and lifetime reproductive success. However, few studies have yet examined the extent and causes of variation in dominance tenure and the duration of breeding lifespans. Here, we investigate the processes that terminate dominance tenures and examine how they differ between the sexes in wild Kalahari meerkats (*Suricata suricatta*), a cooperative breeder where a dominant breeding pair produces most of the young recruited into each group. Mortality and displacement by resident subordinate competitors were important forms of dominance loss for both sexes. However, dominant males (but rarely females) were also at risk of takeovers by extra-group invading males. Dominant males also differed from dominant females in that they abandoned their group after the death of their breeding partner, when no other breeding opportunities were present, whereas dominant females that lost their partner remained and continued to breed in the same group. We show that a larger number of processes can terminate dominance tenure in males with the result that the average male tenure of breeding positions was shorter than that of females, which contributes to the reduced variance in the lifetime reproductive success in males compared to females. Our analysis suggests that sex differences in emigration and immigration may often have downstream consequences for sex differences in reproductive variance and for the selection pressures operating on females and males.

## Teaser

For many social species, maintaining long tenures in dominant breeding positions is an important determinant of lifetime reproductive success. In meerkats, dominant males have shorter tenures than females, resulting in lower and less variable lifetime reproductive success compared to females. We show this contrast in tenure length between the sexes occurs because male meerkats lose dominance in more varied ways than females.

## INTRODUCTION

In many group-living animals, access to reproduction is influenced by social status and dominant individuals often benefit from increased reproductive rates (Solomon and French 1997; Alberts 2012; Pusey 2012; Koenig and Dickinson 2016) and, in some species, from increased survival, too (Dammann et al. 2011; Cram et al. 2018; Snyder-Mackler et al. 2020). As a result, individuals that acquire high social status often have substantially higher lifetime reproductive success than those that do not (Shivani et al. 2022). A substantial number of studies of social mammals have consequently explored the factors that predict the acquisition of dominance status and suggest that age (Archie et al. 2006), body mass (Veiberg et al. 2004), weapon size (Berger and Cunningham 1998), and the level of social support from other group members (Bissonnette et al. 2015; Vullioud et al. 2019) can all be important. However, the fitness benefits of high social status can also depend on the duration of time over which dominant individuals maintain their high status, and the factors that affect the tenure of dominance positions may differ from those that control the acquisition of high status (Clutton-Brock 1988; Clutton-Brock et al. 2006; Robbins et al. 2011; Pusey 2012; Lardy et al. 2015). As yet, only a few studies have investigated the social and ecological processes that terminate dominance tenures and the extent to which individuals adopt strategies that help to prolong their tenure of high status and the duration of their breeding lifespans (Lukas and Clutton-Brock 2014; Port et al. 2017).

Long-term studies of group-living mammals demonstrate that dominance tenures commonly end in one of four ways. In some cases, mortality ends most tenures with dominant individuals maintaining their status throughout adulthood until death (Rood 1990; Sharp and Clutton-Brock 2010). In others, individuals are deposed from dominance, either by within-group competitors who challenge and displace them, or by extra-group individuals of the same sex who invade and take over the group (Jack and Fedigan 2004; Teichroeb and Jack 2017). Finally, dominants may also abandon their position to undergo secondary dispersal, which is often associated with attempts to gain or increase access to breeding opportunities (Packer and Pusey 1987; Hidalgo Aranzamendi et al. 2016). The relative frequency of these four processes often differs between species and the sexes (Rood 1990; Clutton-Brock 2016; Teichroeb and Jack 2017), which can lead to sex differences in dominance tenure, in the length of breeding lifespans and in the extent of variance in lifetime reproductive success (Clutton-Brock 1989; Lukas and Clutton-Brock 2014).

In some mammals, sex differences in dominance tenure appear to be a consequence of sex differences in mating competition: for example, in many polygynous species, dominant males have shorter tenures and shorter breeding lifespans than females because intense mating competition between males increases the turnover of dominants (Promislow 1992; Clutton-Brock and Isvaran 2007; Lukas and Clutton-Brock 2014). In other cases, sex differences in rates of turnover among breeding individuals appear to be a consequence of sex differences in survival associated with differences in parental care (Liker and Székely 2005; Santos and Nakagawa 2012). However, sex differences in dominance tenure and in the duration of breeding lifespans also occur in species where there are no obvious sex differences either in mating competition or in parental care. For example, in some cooperative breeders, including Damaraland mole-rats, *Fukomys damarensis*, and meerkats, *Suricata suricatta*, males have shorter dominance tenures than females (Clutton-Brock et al. 2006; Young and Bennett 2013) although breeding is largely confined to a single breeding pair within each group and both sexes are involved in the care of offspring (Hauber and Lacey 2005; Clutton-Brock and Manser 2016; Zöttl et al. 2016).

Here, we describe the mechanisms that constrain the dominance tenures and breeding lifespans of both sexes in wild Kalahari meerkats and investigate the reasons why dominant females have longer dominance tenures than dominant males. We identify the different ways in which dominant female and male meerkats lose their breeding positions, examine the factors that influence them, and compare the relative frequency with which they terminate dominance tenures. Meerkats are desert-adapted mongooses that live in stable groups of 15 individuals on average, consisting of a single dominant breeding pair and several generations of subordinates (Griffin et al. 2003). Only a small proportion of the population ever acquires dominance and those that do commonly produce over 80% of the offspring born into the group (Hodge et al. 2008; Spong et al. 2008). Female dominants achieve this reproductive monopoly by suppressing subordinate female fertility, evicting pregnant subordinates from the group, and killing any offspring they produce (Clutton-Brock, Brotherton, et al. 1998; O’Riain et al. 2000; Young et al. 2006). By contrast, male dominants appear to largely exclude male competitors from mating by guarding the dominant female when she is in estrus (Spong et al. 2008). Dominant individuals also experience lower mortality than subordinates and have longer lifespans (Cram et al. 2018), during which they can produce multiple litters. As a result, the duration of their dominance is an important determinant of their direct fitness (Clutton-Brock et al. 2006), accounting for 41% and 55% of the variance in reproductive success for males and females, respectively (Hodge et al. 2008; Spong et al. 2008).

Female meerkats may acquire dominance in their natal group or following dispersal: either by inheriting the position following the death of the previous incumbent, by displacing the current dominant, or by dispersing to found a new group where they claim dominance (Hodge et al. 2008; Duncan et al. 2018). Subordinate females rarely leave their groups voluntarily, and dispersal is the result of repeated evictions by the dominant female (Clutton-Brock, Brotherton, et al. 1998), which eventually results in their permanent emigration (Maag et al. 2018). Evicted females that leave the territory of their group are chased away by the members of other groups and are seldom able to join them or to evict their resident females, therefore to avoid the mortality risks associated with extra-group movement they must either form a new group with dispersing males or return to their previous group (Cram et al. 2018; Maag et al. 2018). Following the acquisition of dominance, females experience a period of accelerated growth (Huchard et al. 2016), in addition to undergoing marked changes in their morphology (Russell et al. 2004) and hormone physiology (Carlson et al. 2004; Davies et al. 2016).

The life histories of male meerkats follow a different course. As female meerkats avoid mating with familiar males in their natal groups (Griffin et al. 2003; Spence-Jones et al. 2021), males must disperse from their birth groups to search for breeding opportunities in other groups, often forming dispersing coalitions with other males in their group, who are frequently their siblings (Young et al. 2007; Mares et al. 2014). Males can acquire dominance at a new group formed with dispersing females, or (unlike females) can join established groups, either by filling a dominance vacancy or by forcing out the incumbent dominant male in an external takeover (Spong et al. 2008; Mares et al. 2012). In addition, males can also disperse into a subordinate position and subsequently acquire dominance through inheritance or by displacing the incumbent dominant. As in females, males undergo accelerated growth following the acquisition of dominance. However, dominants of both sexes display senescent declines in body mass in mid-to-late life (Thorley et al. 2020), and they are, therefore, not always the heaviest individuals in their groups. In addition to the risk of losing dominance to takeovers by extra-group intruders, male dominants may also undergo secondary dispersal, often following the death of their breeding partner (Clutton-Brock et al. 2006; Spong et al. 2008). These additional forms of dominance loss could contribute to the shorter tenures of dominant males, and we explore whether this is likely to be the case.

## METHODS

### Study system

Our study was conducted using long-term data collected on a wild population of meerkats living on and around the Kuruman River Reserve in the southern Kalahari Desert, Northern Cape, South Africa (26°58ʹS, 21°49ʹE). Between August 1994 and August 2021, habituated groups of wild meerkats were visited three to four times a week. At any one time between 6–21 groups were followed, with a mean ± SD group size of 15 ± 7 individuals. In addition to the dominant breeding pair and the dependant offspring/juveniles, groups also included several subordinate helpers, consisting of adult females (mean = 3, range = 0–19), adult males (mean = 4, range = 0–24), and sub-adult helpers (6–12 months old) (mean = 3, range = 0–17). Observational data were collected on groups for 3–4 h in the morning following their emergence from the burrow, and for 1 h in the evening before the burrow return. Particular attention was paid to dominance interactions, and changes in dominance were recorded alongside contextual information. At every visit, a range of other data was also collected, including but not limited to group composition, individual pregnancy status, injuries and signs of disease, and interactions with other groups or dispersing/roving individuals (Clutton-Brock and Manser 2016). Most individuals in the population could also be weighed on electronic scales using egg crumbs and water as incentive. At morning visits, an attempt was made to weigh each group member before they started foraging (referred to as “body mass” hereafter).

### Dominance tenure length

Dominant individuals of both sexes are behaviorally distinct. Dominants display agonistic behaviors at higher rates than subordinates, frequently asserting dominance over same-sex group members and receiving ritualized submissions in return (Clutton-Brock et al. 2006; Kutsukake and Clutton-Brock 2006, 2008). Dominants also scent mark at higher frequencies than same-sex subordinates (Jordan 2007), and in females, the incumbent dominant regularly evicts other females in her group (Clutton-Brock, Brotherton, et al. 1998). Dominance was assessed through the observation of social interactions, and the start of an individual’s dominance tenure was set as the point at which an individual was seen asserting dominance and receiving submissions from all other adult same-sex group members. Due to the high frequency of our observations, the start of dominance could usually be detected within 0–3 days of dominance acquisition. To ensure accurate estimations of tenure length, we excluded individuals that were likely to have acquired dominance long before their observed start date—such as the dominants of newly discovered established groups. A dominant’s tenure length was calculated as the time between the start and end of their dominance, the latter being set to the earliest date that they were no longer observed as dominant. Sometimes, a dominance period was disrupted by the brief absence of the dominant individual from the group or due to temporary social instability. However, unless these events resulted in a change of dominance, tenure was considered continuous throughout.

Though males can acquire behavioral dominance in their natal groups, “natal” dominant males rarely breed with resident females there, and the principal benefit of dominance status appears to be that it is associated with an increase in the probability that individuals will subsequently acquire dominance in another group (Spence-Jones et al. 2021). Since the aim of this study was to explore the factors affecting the tenure of dominant breeders of each sex, we consequently did not include tenures where a male acquired behavioral dominance in their natal group in our analyses (*n* = 57). In contrast, females that acquire dominance can successfully breed irrespective of their natal status, so we analyzed both natal and dispersed female dominants. In total, our sample consisted of 225 male and 187 female dominance tenures with accurate start dates, in 70 different established groups, representing 182 unique males and 176 unique females. Of these, 212 male and 166 female tenures covered complete bouts of dominance with known end date. All remaining tenures were right censored in our analyses because the dominants still held their position at the end of the study, or because observation of their group ceased before they lost their position.

### The forms of dominance loss

To identify the events associated with dominance loss, we used a combination of data sources to provide context for the tenure end, including researcher field logs, life history records, individual health logs, and inter-group movement data. Based on these data, we identified the loss of dominance as falling into one of six forms: (1) *mortality*, (2) *internal displacement*, (3) *external takeovers*, (4) *abandonment*, (5) *group disintegration*, and (6) *disappearance*. Changes in dominance status could be identified and characterized either by direct observation of the event or by observations of changes in the membership and dominance relationships of the group (284/378). However, for a subset of tenures, the circumstances of dominance loss could not be identified definitively, and the form of loss was instead inferred from contextual information (58/378). A detailed breakdown of category assignments is presented in the Supplementary Material (SI 1), but a brief classification is as follows: (1) in-situ *mortality* could be identified by the discovery of the body/radio-collar or where dominants were euthanized during the late stages of an infection with a meerkat-specific strain of Tuberculosis (TB), *Mycobacterium surricattae* (Parsons et al. 2013), a terminal disease where individuals commonly die within 6 months of developing clinical signs (Patterson et al. 2017). *Mortality* was also assumed when dominants were last seen with obvious signs of clinical disease or were in an emaciated state indicative of a terminal decline. (2) *Internal displacement* occurred when a dominant was overthrown by a same-sex resident subordinate, which was either directly observed or inferred by the presence of a rank switch following a period where the group was not observed. (3) *External takeovers* were identified as cases where non-resident individuals from other groups migrated into the group, often in coalitions, and replaced the incumbent dominant. When not directly observed, we inferred takeovers from the presence of a new immigrant dominant. (4) *Abandonment* occurred when the dominant actively abandoned their status and left their group permanently or for an extended period to attempt secondary dispersal. *Abandonment* could be identified by observations of the dominant leaving the group, roving at other groups following disappearance from their own group, or by the disappearance of multiple males at the same time, indicative of a dispersing coalition. (5) *Group disintegration* was assigned to dominants whose tenures ended following the failure of their group. While group disintegration was accounted for within our analyses, group failure in our study population has recently been investigated elsewhere (Duncan et al. 2021), therefore, we did not explicitly analyze its causes here.

Finally, there was a subset of dominants who *disappeared* (6) while their group was not under observation and for whom there was no additional information that allowed the form of dominance loss to be accurately inferred (males = 23, females = 13). In females, it is rare for dominants to abandon their status or experience takeovers, and they often remain resident following internal displacement. Therefore, disappearance is likely to represent *mortality* in females, so for descriptive analyses, we consider female dominants that disappeared as having died. In contrast, males experience multiple forms of tenure loss in non-negligible frequencies, which makes it impossible to accurately infer the circumstances of tenure loss for male dominants that disappeared. Therefore, when analyzing the frequencies of the different forms of male tenure loss, we excluded disappeared dominants. As the disappearance of male dominants is most likely to be associated with abandonment and mortality, our estimates of the frequency of abandonment and mortality are likely to be slightly underestimated, while external takeovers and internal displacements will be slightly overestimated.

### Statistical analyses

To capture general patterns of dominance loss across time, we first collapsed the different forms of dominance loss together and modeled tenure duration using parametric survival models. The sexes were first modeled independently to identify the best-fitting survival distribution (Supplementary Material SI 2), before being modeled together—the latter allowing for sex differences in tenure length to be compared statistically. Subsequently, we conducted competing risk analyses on each sex to model the different “forms” of dominance loss together. Competing risk models capture situations where two or more “modes of failure” compete to end the life of a system, which in our case is the tenure of dominants. Survival analyses that consider only a single mode of failure can generate estimation bias by truncating or censoring individuals with a known end date but an unknown cause of failure. Competing risk models correct for these biases by estimating the marginal probability of all failure modes within a single framework.

In our models, individuals transition from a state of dominance to a state of lost dominance, with each form of tenure loss being treated as a separate absorbing state. Royston-Parmar spline models were used for the competing risk analyses to capture temporal variation in the probability of particular forms of dominance loss occurring. Model selection was guided by Akaike's information criterion (AIC), and plots of the predicted survival and hazard curves alongside the raw data were used to confirm the goodness of fit. As not all forms of tenure loss were present in statistically analyzable frequencies in both sexes, we fitted separate models for each sex. However, in cases where any given form of tenure loss was present in substantial frequencies in both sexes (mortality and internal displacement), we fitted cumulative incidence functions and Cox proportional hazard models on both sexes jointly to directly compare patterns of dominance loss. As disappeared dominants were unlikely to represent internal displacements in both sexes, we accounted for disappeared dominants as a competing risk when comparing displacement rates between the sexes. However, when comparing mortality between the sexes, we report models both truncating male dominants that disappeared, and with disappearances representing mortality events, as in females.

### Factors associated with the different forms of dominance loss

To investigate whether individual and social factors predicted the different forms of dominance loss, we fitted time-varying semi-parametric Cox proportional hazard models. Both male and female dominants who disappeared were accounted for in these models, but no assumptions were made about how they lost dominance to avoid unintended bias. We also accounted for tenures ended by group failure, but we did not explicitly test any covariates on this form of dominance loss. Competing risk models allow for the fitting of transition-specific covariates, such that the strength and direction of any fitted covariate effect (e.g., group size) can differ according to the transition. In these analyses, all covariates were fitted as transition-specific and were only estimated for transitions where we hypothesized an effect a priori, with full models reported. An assumption of the Cox-proportional hazards model is that covariates are proportional, meaning their effect remains constant across time. Where necessary, we controlled for covariates that violated this assumption by fitting an interaction with time (*time*:covariate, Fox and Weisberg 2002). For comparability of effect sizes, all variables were mean centered, with continuous variables additionally divided by 2 standard deviations (Gelman 2008).

Dynamic changes in weather and group composition can occur over short-time scales in our study population. Therefore, to allow for covariates to temporally vary across tenures in our models, we divided dominance tenures into 1-month periods and calculated covariates as monthly means. Covariates included body mass, body mass^2^, total group size (number of individuals older than 6 months), and the number of same-sex subordinate adults (older than 1 year of age); the latter to investigate the potential effect of within-group competitors on internal displacement and external takeovers. For males, we further differentiated subordinate adults into natal subordinates who were born in the group and immigrant adults who had migrated into the group (usually with the dominant male), under the expectation that the competitive environment faced by dominants varies according to the origin of the subordinate males (and thus their relatedness to dominant female). In this case, we included both terms and compared the model fit (AIC) to a model in which subordinate adult male count was included as a single term. Predictions from the competing risk model were then used to characterize the combined effect of subordinate competitors on different causes of tenure loss, and additional survival analyses of their effect on the overall probability of dominance loss were conducted (Supplementary Material SI 6). For females, to remove the possibility that weight gain during pregnancy could confound general body mass effects, we repeated our analyses on a data set that excluded weights taken during periods of visible pregnancy (results are reported in the Supplementary Material, SI 3). For modeling mortality in both sexes, we included a binary term noting if the dominant individual’s breeding partner had died within the current or previous three months of their tenure, and for female internal displacement, we included a term indicating whether foreign males had migrated into the group either during the current or previous month of their tenure was fitted. Finally, for male abandonment, we included a binary term noting whether there were still resident females who were born before the dominant male immigrated into the group (“unfamiliar females”), as well as a binary term noting whether a male had lost their breeding partner (mortality or disappearance) within the current or last three months of their tenure. After excluding monthly periods with missing data, the sample for males consisted of 2555 one-month periods from 200 dominance bouts with 180 observed tenure ends. For females, there were 3151 one-month periods from 178 dominance bouts with 149 observed tenure ends. The absence of body mass measurements accounted for most of the excluded periods. To ensure that the estimation of social effects used as much of the full dataset as possible, we also re-ran cause-specific hazard models without body mass (Supplementary Material SI 4) and used these models to predict the effects of group size and composition.

We conducted all analyses within the R statistical environment (R Core-Team 2016), R version 4.2.0. To characterize the dominance tenures of both sexes, we fitted semi-parametric and parametric survival models in the R packages *Survival* (Therneau 2023) and *flexsurv* (Jackson 2016). To investigate the different forms of tenure loss, we used cumulative incidence functions and competing risk analyses, a type of multi-state modeling, using the R package *mstate* (Wreede et al. 2011).

## RESULTS

**Figure 1:**
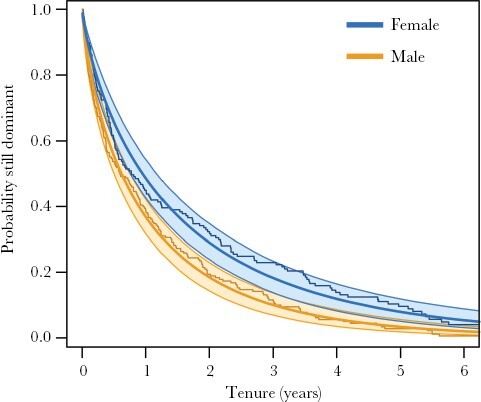
The probability of male (orange) and female (blue) dominants holding their position over time. Plotted survival curves (solid line) and accompanying confidence intervals (shaded ribbons) were predicted from a parametric survival model fitted with a Weibull distribution where both the scale and shape parameters were allowed to vary in relation to sex. The predictions are overlaid on raw Kaplan–Meier plots (darker lines) for each sex. The x-axis is restricted to 6 years to allow easier visual comparison between males and females (some females held tenures for over ten years).

Male dominants had significantly shorter tenures than females (Figure 1, scale parameter: mean effect [95% CI] = −0.416 [−0.689, −0.143]), with mean male tenure length 12.7 months (median = 6.3, SD = 15.2, range = 0.1–68.3 months) and mean female tenure length 18.1 months (median = 7.3, SD = 22.5, range = 0.2–127.9 months). However, the shape of dominance loss over time did not differ between the sexes (shape parameter: mean effect [95% CI] = −0.026 [−0.186, 0.133]), with the instantaneous risk of losing dominance highest early in tenure and declining as tenures progressed (Weibull shape parameter <1, *k* = 0.78; Figure 1).

A few individuals of both sexes experienced multiple temporally distinct periods of dominance over their lifespan, either in the same group or in different groups. Males were more likely to hold multiple distinct positions of dominance over their lifespan (Proportions Test: *N*_1_ = 156, *N*_2_ = 171, χ^2^ = 11.6, *P* < 0.001): 20% of male dominants held multiple positions versus 6% of female dominants. No females were dominant on more than two occasions, whereas two males held four periods of dominance in multiple groups. Even accounting for repeated periods of dominance, males tended to spend less time in dominance across their lifespan overall than females (male mean = 15.7 months, female mean = 19.3 months), although not significantly so (Linear Model Log Transformed: Estimate [95% CI] = −0.215 [−0.544,0.114], *P* = 0.2).

### Dominant mortality

**Figure 2:**
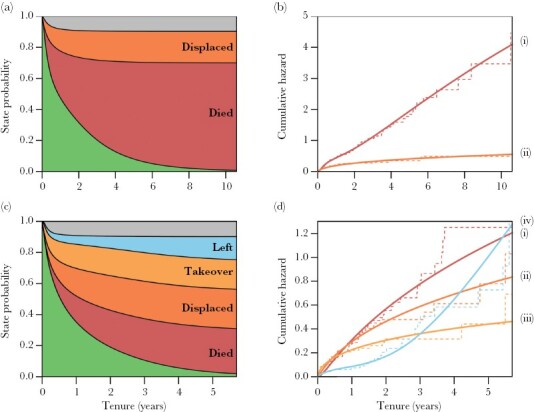
Routes to tenure loss in male and female meerkats. Stacked state probability (a,c) and cumulative hazard (b,d) plots for female (a,b) and male (c,d) dominants. Individuals start in a position of dominance (green) and transition to a specific state when their tenure ends, representing mortality (i, red), internal displacement (ii, dark orange), external takeovers (iii, light orange), abandonment (iv, blue) or other forms (gray). The width of a band represents the predicted proportion of individuals who have lost their dominance via a specific form each time step. The cumulative hazard predictions (solid lines) are plotted over the raw data (dashed lines) and indicate the change in risk of a particular form over time, with straight lines indicating constant risk and curvature indicating either increasing risk (exponential) or decreasing risk (asymptotes), respectively. All plots were produced with predictions from competing risk models fitted with parametric Royston-Parmar spline models with the sexes modelled independently.

**Figure 3:**
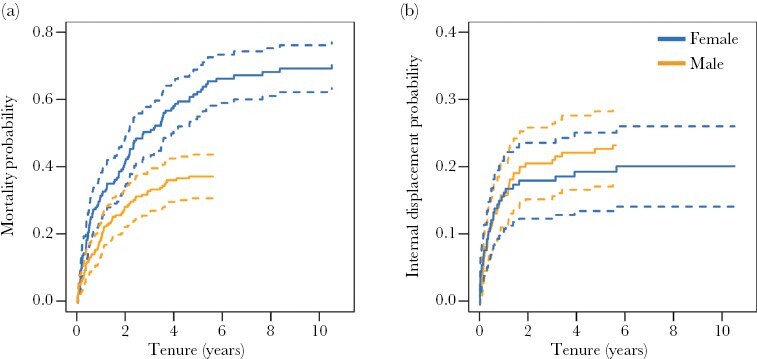
Cumulative incidence plots for dominance loss through mortality (a) and internal displacement (b) with the data grouped by sex, males in orange and females in blue. Solid lines display the cumulative incidence and dashed lines represent the 95% confidence intervals. State probabilities were calculated from nonparametric cumulative incidence functions using the “Cuminc” function in the *mstate* package.

Mortality was the most prevalent form of dominance loss in both sexes (Figure 2a,c) and was responsible for 68.7% of tenure ends in females (114/166) and 29.1% in males (55/189). For both sexes, the mortality hazard was approximately constant across tenure (Figure 2b,d). Despite females generally having longer tenures, Cox proportional hazard models and cumulative incidence plots indicated they had higher mortality rates than dominant males (Estimate ± SE = −0.441 ± 0.167, *P* = 0.008, Figure 3a). This sex difference is probably exaggerated by our decision to truncate the tenures of males that disappeared, which will underestimate the total contribution of mortality in dominant males. Nevertheless, if we instead assume that disappeared dominant males represent mortality (itself likely an overestimate), dominant females still tended to show higher mortality than dominant males (Estimate ± SE = −0.230 ± 0.150, *P* = 0.12).

The risk of mortality declined with increasing body mass in both sexes. This effect took a quadratic form with the benefits of increasing body mass declining as mass increased. For females, the effect also began to reverse at the higher end of the mass distribution such that the heaviest females experienced an increased mortality risk relative to females of mean body mass (Supplementary Material SI 5). As high body masses were only reached by heavily pregnant females, this suggests a possible mortality cost of pregnancy which may also contribute to the higher mortality rate observed in female dominants. In both sexes, the risk of mortality also increased if the dominant’s partner had died in the previous three months (Table 1). One possibility here is that this reflects cases where both dominants were concurrently infected with TB, which can infect whole groups and drive them to extinction (Patterson et al. 2017; Duncan et al. 2021).

**Table 1:** Outputs for cause-specific competing risk models of tenure loss in male and female dominants

	Males					Females			
	Estimate ± SE	HR	z-value	*P*		Estimate ± SE	HR	z-value	*P*
Mortality (*N* = 50)					Mortality (*N* = 90)				
Body Mass	−0.539 ± 0.290	0.58	1.86	0.063	Body mass	−0.700 ± 0.186	0.50	3.76	<0.001
Body mass^2^	0.789 ± 0.273	2.20	2.89	0.004	Body mass^2^	0.925 ± 0.172	2.52	5.39	<0.001
Group size	−0.577 ± 0.370	0.56	1.56	0.120	Group size	−0.706 ± 0.311	0.49	2.31	0.021
Partner death	0.832 ± 0.384	2.30	2.17	0.030	Partner death	0.817 ± 0.311	2.26	2.63	0.009
Internal displacement (*N* = 44)					Internal displacement (*N* = 31)				
Body mass	−1.640 ± 0.436	0.20	3.76	<0.001	Body mass	−0.233 ± 0.308	0.79	0.76	0.450
Immigrant males	0.898 ± 0.148	2.45	6.07	<0.001	Body mass^2^	0.587 ± 0.299	1.80	1.96	0.050
Natal males	0.264 ± 0.400	1.30	0.66	0.508	Adult females	0.885 ± 0.375	2.42	2.36	0.018
*time:*Body mass	0.588 ± 0.290	1.80	2.03	0.043	Immigration event	1.174 ± 0.426	3.23	2.75	0.006
External takeover (*N* = 28)									
Body mass	−1.255 ± 0.355	0.29	3.54	<0.001					
Immigrant males	−2.558 ± 0.830	0.08	3.08	0.002					
Natal males	−1.142 ± 0.837	0.32	1.37	0.172					
Abandonment (*N* = 25)									
Partner loss	1.000 ± 0.539	2.73	1.86	0.063					
Unfamiliar females	−2.603 ± 0.534	0.07	4.88	<0.001					

### Internal displacement

Internal displacement by resident subordinates of the same sex was the second most common form of tenure loss: responsible for 21.1% in females (35/166) and 26.5% of cases in males (50/189). For both sexes, the risk of internal displacement was highest at the start of dominance tenure and declined subsequently (Figure 2b,d). There was no evidence of a sex difference in the overall risk of internal displacement (Estimate ± SE = 0.355 ± 0.223, *P* = 0.112, Figure 3b), but the slight non-proportionality in the hazard for sex suggests that the decline in risk over time may be more pronounced in females (Supplementary Material SI 7).

**Figure 4:**
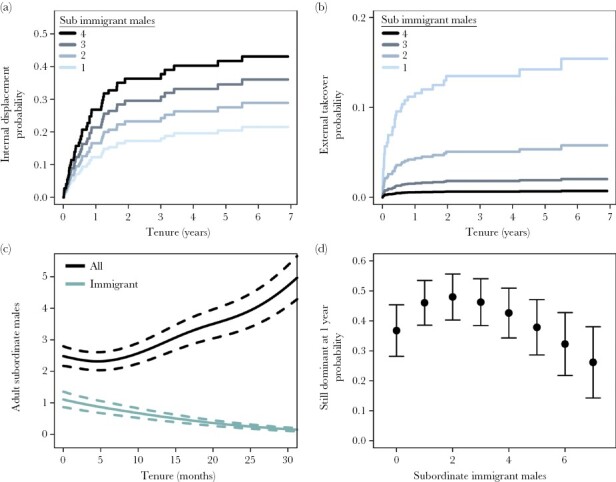
Predictions from a semi-parametric competing risk model showing the effect of the number of subordinate immigrant males (1–4) on the probability of dominant males experiencing internal displacement (a) and external takeover (b). For predictions, all non-displayed covariates are held at their mean, except for the number of natal males which was set to one to better represent the beginning of a dominant male’s tenure where adult natal males were less prevalent. (c) The predicted number of all adult subordinate males (black), and the number of immigrant adult subordinate males (gray), across the first 30 months of male’s tenure (mean ± 95% CI). The numbers are estimated from a generalized additive model with a negative binomial distribution, where tenure ID was fitted as a random effect and tenure time (months) was modelled using splines. (d) The predicted probability that a dominant will still be dominant 1 year into their tenure depending on the number of immigrant subordinate males resident in their group. Predicted means plotted as points with error bars displaying the 95% CI. The range of values plotted was limited to 0–7 subordinate immigrants, as it is rare for dominants to reside with more than seven subordinate immigrants (Supplementary Material SI 6).

Lighter dominants of both sexes were more likely to be internally displaced. For females, the effect of body mass was quadratic so that the benefits of increasing mass became less pronounced as mass increased (Table 1). For males, the body mass was not a proportional hazard, suggesting the strength of the body mass effect decreased across tenure (Table 1). The presence of increasing numbers of same-sex subordinate adults in the group was also associated with an increased risk of internal displacement in both sexes. While dominant females could be internally displaced by any resident subordinate, dominant males were mostly at risk of displacement by resident immigrant subordinate males—individuals who had often immigrated with them into the same group and were frequently their brothers. As a result, displacement risk in males was dependent on the immigrant status of the subordinate males: greater numbers of subordinate immigrant males increased the displacement risk of dominant males (Figure 4a, Table 1), while numbers of natal subordinates had no effect (Table 1). Since the number of resident immigrant subordinate males declined as male tenure progressed (Figure 4c), this may explain why displacement risk also declined across tenure. Dominant females were also significantly more likely to experience internal displacement in the month following the immigration of new males into the group (Table 1), and there was some evidence this effect was weaker at the start of tenure (Supplementary Material SI 8).

### External takeovers

External takeovers by extra-group intruders were very rare in dominant females, with only two recorded cases of a female immigrating into an established group, taking dominance there, and replacing the previous dominant female. In both cases, the groups involved were very small (three to six individuals) and had only one resident female, the dominant. In contrast, external takeovers represented a substantial risk for dominant males with 19.6% of tenures ending with the invasion of extra-group males (37/189). Like internal displacements, the risk of external takeover was highest at the beginning of a dominant male’s tenure and declined as time progressed (Figure 2d) and was more likely for lighter dominants (Table 1). The presence of increasing numbers of subordinate immigrant males reduced the risk of external takeover (Table 1, Figure 4b). Takeover risk also declined as the number of resident subordinate natal males increased, and this effect was statistically significant when modeled using the larger dataset that ignored missing body mass data (Estimate ± SE = −2.01 ± 0.882, *P* = 0.023, Supplementary Material SI 4). Though the numbers of immigrant and natal subordinate males were both associated with a reduced risk of takeover, the per-capita effect of immigrant males was stronger (Estimate ± SE = −1.00 ± 0.270) than that of natal males (Estimate ± SE = −0.306 ± 0.134), and models that discriminated between the two classes of males provided a better fit than models that included the total number of subordinate males as a single term (ΔAIC > 2).

While subordinate immigrant males reduce the risk of external takeovers for dominant males, they increase the risk of internal displacement. Predictions from the competing risk model suggest that the benefit of immigrant males in this context typically outweighs their cost, as the relative risk of internal displacement only exceeds the risk of external takeovers in cases where there are unusually large numbers of immigrant subordinates present in the group (>4, Figure 4d, see also Supplementary Material SI 6). Most of the time, the effect of the number of immigrant subordinate males falls below this threshold, and their presence is expected to prolong male dominance tenures by reducing external takeover risk.

### Abandonment

**Figure 5:**
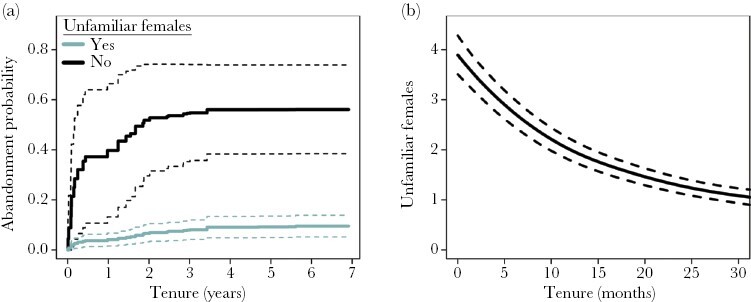
Model predictions (solid line) with 95% confidence intervals showing the effect of the absence of unfamiliar females on the probability of dominant male abandonment (a) and the mean spline for the number of unfamiliar females resident in the group across the tenures of dominant males estimated from a generalized additive model with a negative binomial distribution and tenure ID fitted as a random effect (b). Predictions for the effect of unfamiliar female presence on abandonment were estimated from a semi-parametric competing risk model with all other predictors held at the population average. Visualization of the number of unfamiliar females across tenure were restricted to between 0 and 30 months to allow for clearer visualization of changes in the first few years of dominance.

Dominant females were never observed to abandon their groups voluntarily. In contrast, 14.8% of dominant male tenures ended with the dominant male voluntarily abandoning his group (28/189). The likelihood that dominant males abandoned their position was associated with the kinship structure of the group and the availability of possible breeding partners. Females born prior to the immigration of dominant males (“unfamiliar females”) are typically unrelated to the dominant male and represent possible breeding partners, while females born after the immigration of dominant males (“familiar females”), are likely to be close relatives and avoid mating with familiar dominant males. As would be expected, the probability of abandonment substantially increased when there were no longer any “unfamiliar” females present (Figure 5a; Table 1). Dominants were also more likely to abandon their group after their breeding partner died or disappeared (Table 1, Supplementary Material SI 4), and of the nine abandonments following partner loss, seven were cases where the dominant female that died represented the last resident unfamiliar female. As the tenure of dominant males progressed, the number of resident unfamiliar females declined because they either died or dispersed (Figure 5b), increasing the likelihood of there being no unrelated females to replace dominant females that die. This may explain why the frequency of abandonment increased across the tenure of dominant males (Figure 2d).

## DISCUSSION

In species with high reproductive skew, the length of time that individuals hold positions of dominance often correlates closely with their lifetime reproductive success (Clutton-Brock 1988; Rossiter et al. 2006; Robbins et al. 2011). This is true of many singular cooperative breeders (Hawn et al. 2007; Sparkman et al. 2011; Young and Bennett 2013; Lardy et al. 2015; Fitzpatrick and Bowman 2016), including meerkats (Clutton-Brock et al. 2006). To investigate the factors that affect variation in tenure length in meerkats, our study analyzed the dominance tenures of large numbers of males and females and identified the multiple routes through which dominance can be lost. Our results showed that tenures ended either when dominant individuals died, when they were displaced by resident members of their group or by intruders from other groups, or when they voluntarily abandoned their group. Mortality ended most dominance tenures in both sexes, being responsible for 29% of tenure ends in males and 69% of females. Internal displacement was also prevalent in both sexes, ending 27% of male tenures and 21% of female tenures. However, in contrast to females, a substantial number of dominant males experienced external takeovers by same-sex intruders from other groups (20%), and a sizeable proportion also voluntarily abandoned their position (15%)—usually when breeding opportunities were absent in their group.

In both sexes, low body mass was associated with an increased risk of mortality and of internal displacement by resident group members. For males, low body mass also made external takeover by extra-group intruders more likely. These associations probably reflected the importance of body mass in competitive interactions for meerkats. Previous research on our study population has shown that the heaviest subordinate females are usually most successful in competing for dominance vacancies (Hodge et al. 2008; Duncan et al. 2018), and subordinates of both sexes appear to track and respond to the growth of close competitors in a strategic fashion (Huchard et al. 2016). Body mass is also likely to be a good indicator of a dominant’s condition and is associated with the ability of dominants to suppress subordinate group members (Clutton-Brock et al. 2008). A similar relationship between body mass and competitive ability may occur in cooperatively breeding alpine marmots (*Marmota marmota*) where dominant males and females with high body mass were able to maintain longer dominance tenures (Lardy et al. 2012, 2013). In males of this species, dominance loss was preceded by a decline in body mass which was itself linked to the energetic costs of suppressing same-sex subordinates (Lardy et al. 2012). In a similar vein, the experimental feeding of dominant female meerkats during late-stage pregnancy increased the likelihood of subordinate eviction, providing further evidence that high body condition can help dominants to maintain control (Dubuc et al. 2017).

Our study also found that the risk of mortality to dominants of both sexes declined as the size of their groups increased. Mortality can encompass a variety of processes, but a likely contributing factor is that larger group size dilutes predation risk and improves predator detection in the Kalahari (Clutton-Brock et al. 1999), while also increasing a group’s capacity to defend their range against neighbors (Dyble et al. 2019). Previous work on meerkats has also shown that group size has other wide-ranging benefits for all group members, including improvements in growth and body mass (Clutton-Brock and Manser 2016), which result partially from the lower energetic cost of rearing young in large groups (Clutton-Brock, Gaynor, et al. 1998; Clutton-Brock et al. 2000, 2001).

While the mortality risk of dominants declined with increasing group size, increases in group size were associated with an increase in the number of potential same-sex competitors and the risk of internal displacement in both sexes went up as a result. For males, the risk of internal displacement was dependent upon the motivation of subordinate males to compete for dominance. Specifically, our results found that the number of natal subordinate males did not affect the probability of internal displacement, whereas the number of immigrant subordinate males did. This is presumably because, in contrast to immigrant males, natal individuals are related to the incumbent dominant female and therefore stand to gain no direct reproductive benefits by displacing him, as incestuous mating is avoided in meerkats (O’Riain et al. 2000; Leclaire et al. 2013). In contrast, since all resident subordinate females (who are often natal but are sometimes founders) can breed if they acquire dominance, incumbent dominant females are susceptible to internal displacement from all subordinate females, and this is why their displacement risk increased as the number of adult subordinate females rose. Like males, the motivation of subordinate females to compete also increased when breeding opportunities arose, as evidenced by the heightened displacement risk following the immigration of unfamiliar males. The intensification of female-female competition following male immigration has previously been shown to trigger short-term growth acceleration in subordinate female meerkats (Dubuc and Clutton-Brock 2019).

In contrast to dominant females, a substantial number of dominant male tenures ended due to takeovers by same-sex immigrants that had dispersed from other groups. A dominant male’s risk of external takeover declined as the number of resident adult males in their group increased, and this is probably because subordinate males assist in repelling intruders (Mares et al. 2012), as is observed in several species (Putland and Goldizen 1998; Feh 1999; Radford 2003; Teichroeb and Jack 2017). In meerkats, the number of resident immigrant males had a stronger effect on reducing the risk of external takeovers to males than the number of resident natal males. We suggest that this reflects variation in the relative costs of takeover for the two categories of subordinate male, and thus their motivation to repel intruders. If subordinate males fail to repel a takeover, they are vulnerable to expulsion from the group (Mares et al. 2012), exposing them to the various costs of dispersal (Young and Monfort 2009; Maag et al. 2019). However, immigrant subordinates will also lose access to viable breeding females that they have the potential to mate with and the possibility of inheriting a breeding position following the death of the incumbent dominant (Spong et al. 2008).

Our results suggest that by reducing the risk of external takeover, subordinate immigrant males largely offset the potential threat of displacement that they pose to dominant males. This is because it is only when subordinate immigrants are present in uncommonly large numbers—greater than four individuals—that they negatively impact the ability of the dominant male to maintain tenure. The overall cost of displacement to the male is also likely to be lower than that of external takeover because unlike the latter (Mares et al. 2012), displacement rarely results in permanent eviction, and in most cases, the new dominant male will be a sibling whose reproduction will bring them indirect fitness benefits (Griffin et al. 2003). Furthermore, the presence of subordinate group members has been shown to reduce the prevalence of extra-group paternity (Duncan 2022). In combination, these effects might explain why dominant males tolerate other immigrant males in their group, even if doing so means that they will lose some share of paternity (Spong et al. 2008). Similar benefits of resident subordinate males to dominant males have been observed in other mammals that live in multi-male groups (Henzi et al. 2010; Port et al. 2010; Snyder-Mackler et al. 2012), and theoretical models suggest that effects of this kind can favor their evolution (Port and Cant 2014; Port et al. 2018).

The tenure of dominant males was also affected by the kinship structure of breeding groups. When dominant males first migrated into an established group, they were unfamiliar and unrelated to all resident females. As their tenure progressed, mortality and the eviction of older subordinates (Clutton-Brock et al. 2010), as well as the recruitment of their newly produced offspring, resulted in the proportion of unfamiliar non-kin in the group declining, until eventually, only the dominant female was unrelated to them. As in many other social species living in stable groups where kin of both sexes associate throughout their lifespan, inbreeding avoidance is common (Pusey and Wolf 1996; Pike et al. 2021), and meerkats avoid mating with familiar individuals who are commonly close relatives (O’Riain et al. 2000; Griffin et al. 2003). As a result, there is an increasing probability that as the number of unrelated subordinate females declines across a dominant male's tenure, the death of a dominant female will result in a group where the resident dominant male has no unfamiliar, unrelated females to breed with. This scenario often causes them to abandon dominance and undergo secondary dispersal in search of new breeding opportunities.

The absence of mating opportunities has been shown to stimulate secondary dispersal in other social vertebrates. For example, in baboons, adult females also avoid mating with familiar, related males, and immigrant dominant males commonly disperse from their breeding groups when their daughters reach breeding age and the availability of breeding opportunities in their groups declines (Alberts and Altmann 1995). Similarly, in some cooperatively breeding birds where females are the dispersive sex, the absence of in-group breeding partners has been associated with the abandonment of dominant status by widowed “dominant” females (Hidalgo Aranzamendi et al. 2016; Walters and Garcia 2016). These processes may also explain observations of group collapse following the death of dominant breeders in gray wolves *Canis lupus* (Borg et al. 2015), African wild dogs *Lycaon pictus* (Woodroffe et al. 2020), and Damaraland mole-rats (Jarvis and Bennett 1993). In contrast, abandonment of breeding groups appears to be uncommon in cases where dominants continue to have reproductive opportunities available to them after the loss of their breeding partner, such as through the immigration of opposite-sex individuals (Nelson-Flower et al. 2012) or through continued access to extra-group individuals. This likely explains why female meerkats, unlike males, do not abandon their groups (Young et al. 2007; Mares et al. 2014).

Our research on meerkats emphasizes the far-reaching effects of emigration and immigration on the life histories of both sexes, as well as on the intensity of selection and the evolution of social behavior. In meerkats, sex differences in dispersal tendencies affect the maintenance of dominance tenures in both sexes. The prevalence of abandonment and external takeovers in males but not females are ultimately caused by sex differences in dispersal, which expose dominant males to competition with males from other groups and reduce their access to viable mating partners as unfamiliar females progressively leave the group. Dispersal patterns also influenced competition within groups: while dominant females were vulnerable to internal displacement by any resident subordinate, natal or founder, dominant males were only likely to be displaced by immigrant subordinate males since the absence of female immigration into established groups deprives natal subordinate males of potential breeding partners. Since the rate of internal displacement is similar in both sexes, and in-situ mortality is slightly lower for dominant males, our study suggests that it is the additional forms of dominance loss that are responsible for the shorter tenures of males. The lower variance in lifetime reproductive success in males compared to females is consequently a direct result of reductions in their tenure of dominant status (Clutton-Brock et al. 2006).

The consequences of variation in local emigration and social behavior for individual breeding tenures that we describe suggest that sex-specific patterns of dispersal can lead to the evolution of adaptive sex differences in behavior that are not directly associated with anisogamy, intra-sexual or intersexual competition or with ecological divergence between the sexes (see Wilson 1975). They consequently emphasize the diversity of evolutionary processes that underly the evolution of sex differences in behavior, physiology, and morphology, and they emphasize the need to understand the reasons for sex differences in local immigration and emigration (Mabry et al. 2013; Trochet et al. 2016; Li and Kokko 2019). Why, for example, do females frequently remain and breed in their birth groups and males disperse in many social mammals (Lawson Handley and Perrin 2007), while females typically disperse and males remain philopatric in most group-living birds (Greenwood 1980) and a minority of mammalian species (e.g., social equids [Monard et al. 1996], several tropical bats [Moussy et al. 2013], and some primates [Isbell and Jack 2009], including the African apes [Harcourt and Stewart 2007; Stumpf et al. 2009])? One suggestion is that females habitually disperse from their birth groups where the usual duration of the breeding lifespans of males or male kin groups exceeds the age at which female offspring are ready to breed, while they remain and breed in their birth groups and avoid mating with familiar males where the breeding lifespans of males are shorter than the age at which females usually begin to breed, and some comparative evidence supports this (Clutton-Brock 1989; Clutton-Brock and Lukas 2012), but more work is needed to understand the factors that extend or constrain male breeding lifespans.

Overall, our study highlights the benefit of applying a comprehensive analytical approach to breeding lifespans that considers all the ways in which breeding lifespans can start and end. In singular cooperative breeders like the meerkat, breeding tenure is directly tied to dominance, and to understand what makes some individuals reproductively successful therefore requires understanding not only how dominance is acquired, but how it is maintained. Our work emphasizes that intrinsic, demographic, and stochastic processes can all affect the ability and incentive of individuals to maintain their breeding tenures. The relative importance of these processes for the maintenance of tenure will undoubtedly vary across breeding systems and will have consequences for the life histories of males and females and the dynamics of social groups. Future research should explore this variation across breeding systems in greater detail.

## SUPPLEMENTARY MATERIAL

Supplementary material can be found at http://www.beheco.oxfordjournals.org/

arad066_suppl_Supplementary_Material

## FUNDING

The long-term research on meerkats is currently supported by funding from the European Research Council (ERC) under the European Union’s Horizon 2020 research and innovation program (no. 742808 and no. 294494) and a grant from the Natural Environment Research Council (grant NE/G006822/1) to T.C.-B as well as by grants from the University of Zurich to M.B.M. and the MAVA Foundation.

## Data Availability

Analyses reported in this article can be reproduced using data provided by Duncan et al (2023).

## Notes

We are grateful to the Kalahari Research Trust and the Kalahari Meerkat Project for access to facilities and habituated animals on the Kuruman River Reserve, and the surrounding farmers for being able to use their land. We thank the managers, volunteers, students, and researchers who have all contributed to the collection and curation of the long-term data over the decades; in particular, David Gaynor and Tim Vink for organization of the project and databases. We are also grateful to all the members of the Large Animal Research Group for their support and insightful discussions. We thank the Mammal Research Institute and the ethics committee at Pretoria University for their support in logistics and ethics permits, as well as the Northern Cape Department of Environment and Nature Conservation for permission to conduct the research (FAUNA 1020/2016). Long-term data collected within the course of this study fall under the permission of the ethics committee of Pretoria University and the Northern Cape Conservation Service, South Africa (EC011-10, EC048-12, EC010-13, EC031-13, EC047-16) and were carried out adhering to the approved guidelines in these permits, in addition to the standards outlined in the ASAB/ABS (2012) Guidelines for the Treatment of Animals in Behavioural Research and Teaching.
