# Supplementary material for: Dominance loss and tenure maintenance in Kalahari meerkats
Source: Behav Ecol. 2023 Aug 22;34(6):979–91. doi: 10.1093/beheco/arad066 (PMC10636735; doi:10.1093/beheco/arad066)
Supplement: arad066_suppl_Supplementary_Material [file arad066_suppl_supplementary_material.docx]

# Supplementary Materials

## SI 1: Definitions of tenure loss

**
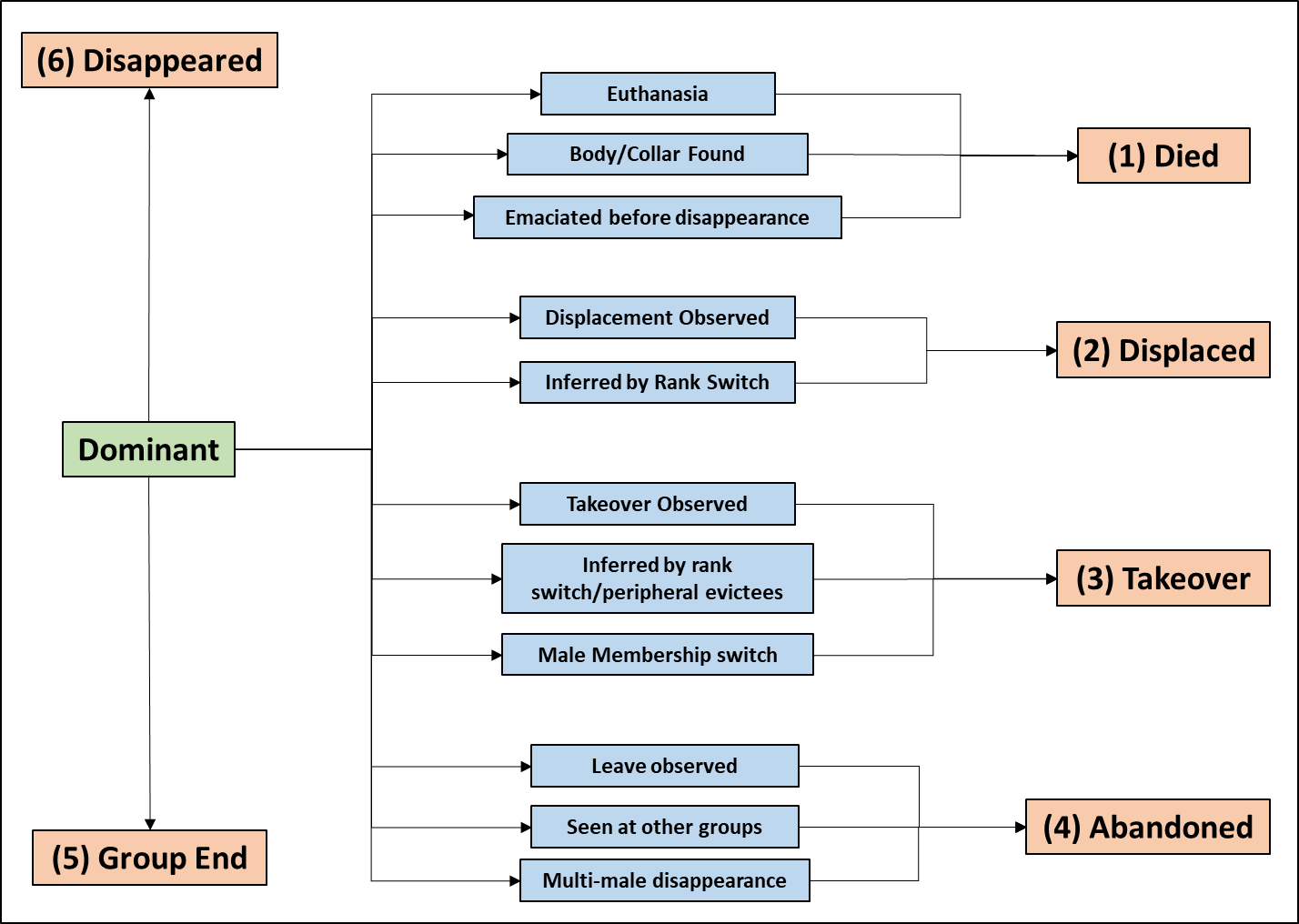
**This supplementary section details the criteria by which the different tenure loss forms of dominants was classified (Figure SI1.1). Due to the incorporation of an additional two years of data, and the re-classification of tenure ends using additional datasets and inference rules to improve our inferences of tenure loss causes, the frequencies of dominance loss reported here differ marginally from (Duncan 2022). However, the results and conclusions are not qualitatively different.

**Figure SI1:** Flow diagram of the different forms of dominance loss investigated in this paper.

*1] Mortality*

While mortality was rarely directly observed, dominant individuals were often radio collared allowing for bodies to be found and mortality confirmed following death by predation or another stochastic cause (N = 77). Tuberculosis is also endemic within the population, and the project has operated a protocol of euthanising individuals in the final terminal stages of the disease, dominants that were euthanised we considered to have lost tenure to mortality (N = 44). Finally, where individuals disappeared in a diseased or emaciated state, but no body was found, we considered these individuals to have died and their tenures ended by mortality (N = 35). In some cases, these terminally affiliated individuals could no longer maintain residency in their groups prior to mortality (usually a day or two before death), and unless, these individuals were observed being forced from the group by conspecifics, we considered their tenure loss to be a function of their impending mortality.

*2] Internal Displacement*

Displacements were often observed (N = 61), with resident subordinate(s) challenging and overthrowing the incumbent dominant. However, displacements could also be accurately inferred by the observation of rank-reversal, where a previously subordinate resident was observed acting dominant and the previous dominant acting as a resident subordinate or being excluded on the periphery of the group, following a period that the group wasn’t observed (N = 24). Howe

*3] External Takeovers*

Takeovers were also directly observed, with out-of-group individual(s) forcibly invading the group and potentially evicting same-sex adult residents, including the incumbent dominant (N = 17). Takeovers could also be accurately inferred following a period of no observation at the group, where new immigrant individual(s) were resident in the group acting dominant, with the incumbent dominant either resident or on the periphery of the group (N = 13). In addition, where the group was visited and new migrant dominants were present and the previous dominant was no long in or within the vicinity of the group, we assumed a takeover had occurred (N = 9). In all but one case, observational gaps were two or fewer days, so while it is possible that the incumbent dominant abandoned their position and new males migrated in after the fact, we suspect a takeover is a more probable cause, as immigrations following observed male abandonment usually take much longer than two days.

*4] Abandonment*

Abandonment was assigned when dominants left their groups permanently or for a substantial period, likely attempting to undergo secondary dispersal, which was confirmed either by the observation of them leaving their group (N = 11), or by their observation elsewhere in the population following their disappearance from their resident group (N = 12). In addition, where a dominant disappeared from the group with other same-sex residents and was not seen again, unless there was evidence of takeover (see above), we classified the dominant as having left (N = 5).

*5] Group Disintegration*

Where a dominants tenure ended because of the group ceasing to exist, the dominants tenure end cause was assigned as group disintegration. This occurred either due to all members of the opposite sex dying (N = 9), or due to fission between the sexes. In many cases fission could be confirmed by observation of same-sex dispersing units following fission (N = 16), however, in the case of 6 groups, disintegration was inferred by finding the resident males by themselves with the females missing, and never being seen again in all but one case. It is possible some of these could represent a takeover, as all resident males were displaced from the group in some observed male takeovers (8/37), however, without information on the fate of the females to confirm the continuation of their tenures and that the males experienced a takeover, we assume these tenures to have ended by group failure (Males = 6, Females = 3).

*6] Disappearance*

With the above classifications assigned, there was still a subset of sample who could not clearly be assigned a tenure loss cause. These were dominants who disappeared by themselves from their groups with no sign of illness. In most of these cases dominance was claimed by a resident individual, and where new immigrants did claim dominance, they did not immediately migrate following the dominants disappearance, suggesting these were not takeovers. In addition, we suspect these disappearances are unlikely to be displacements as overt aggression was not reported and it is uncommon for the incumbent dominant to permanently leave the group immediately following a displacement. As female dominants were not observed to abandon their positions, it is therefore likely these disappearances represent unobserved mortalities and accordingly we treat them as such. However, for males where abandonment can cause tenures to end, it is harder to confidently assume mortality, as potentially the dominant male could have dispersed outside of our population, hence why they are not seen again. Therefore, to avoid substantial bias in the male sample we truncate these dominants from our sample, although this means that the proportion of mortalities and abandonments in males are likely to be slightly under-estimated.

**SI 2:** AIC and Log-Likelihood outputs for parametric survival models fitted with different distributions, modelling dominant tenure lengths in both male and female meerkats, independently and together.

|  | Males | | |  | Females | | |  | Both Sexes | | |
| --- | --- | --- | --- | --- | --- | --- | --- | --- | --- | --- | --- |
| Distribution | AIC | LogLik | ΔAIC |  | AIC | LogLik | ΔAIC |  | AIC | LogLik | ΔAIC |
|  |  |  |  |  |  |  |  |  |  |  |  |
| Weibull | 2958.48 | -1477.24 | 0.00 |  | 2451.03 | -1223.51 | 0.00 |  | 5414.56 | -2705.28 | 0.00 |
| Gompertz | 2976.81 | -1486.40 | 18.32 |  | 2461.27 | -1228.63 | 10.24 |  | 5443.50 | -2719.75 | 28.94 |
| Log Normal | 2973.61 | -1484.81 | 15.12 |  | 2453.27 | -1224.64 | 2.24 |  | 5531.46 | -2713.73 | 16.90 |
| Log Logistic | 2980.41 | -1488.20 | 21.92 |  | 2459.78 | -1227.89 | 8.75 |  | 5442.60 | -2719.30 | 28.04 |
| Gamma | 2959.89 | -1477.94 | 1.40 |  | 2453.54 | -1224.77 | 2.51 |  | 5419.71 | -2707.85 | 5.15 |
| Exponential | 2985.80 | -1491.90 | 27.31 |  | 2468.33 | -1233.16 | 17.30 |  | 5466.53 | -2732.26 | 51.97 |
|  |  |  |  |  |  |  |  |  |  |  |  |

**SI 3:** Competing risk model outputs for females where the body mass measures have been restricted to weights where the dominant female was not visibly pregnancy.

|  | Females | | | |
| --- | --- | --- | --- | --- |
|  | Estimate ± SE | HR | z-value | p |
| *Death (N=79)* |  |  |  |  |
| Body Mass | -1.009 ± 0.270 | 0.37 | 3.73 | <0.001 |
| Body Mass^2^ | 0.582 ± 0.201 | 1.79 | 2.90 | 0.003 |
| Group Size | -0.690 ± 0.329 | 0.50 | 2.09 | 0.036 |
| Partner Death | 0.579 ± 0.345 | 1.78 | 1.68 | 0.093 |
|  |  |  |  |  |
| *Internal Displacement (N=27)* |  |  |  |  |
| Body Mass | -0.740 ± 0.376 | 0.48 | 1.96 | 0.050 |
| Body Mass^2^ | NA | NA | NA | NA |
| Adult Females | 0.918 ± 0.380 | 2.50 | 2.42 | 0.016 |
| Immigration Event | 1.297 ± 0.452 | 3.66 | 2.87 | 0.004 |

**SI 4:** Competing risk model output with body mass not included, resulting in an increased sample size.

|  | Males | | | |  |  | Females | | | |
| --- | --- | --- | --- | --- | --- | --- | --- | --- | --- | --- |
|  | Estimate ± SE | HR | z-value | P |  |  | Estimate ± SE | HR | z-value | p |
| *Death (N=54)* |  |  |  |  |  | *Death (N=94)* |  |  |  |  |
| Group Size | -0.593 ± 0.358 | 0.55 | 1.66 | 0.097 |  | Group Size | -0.912 ± 0.295 | 0.40 | 3.09 | 0.002 |
| Partner Death | 1.071 ± 0.345 | 2.92 | 3.11 | 0.002 |  | Partner Death | 1.210 ± 0.293 | 3.35 | 4.13 | <0.001 |
|  |  |  |  |  |  |  |  |  |  |  |
| *Internal Displacement (N=46)* |  |  |  |  |  | *Internal Displacement (N=31)* |  |  |  |  |
| Immigrant Males | 0.782 ± 0.131 | 2.19 | 6.00 | <0.001 |  | Adult Females | 0.794 ± 0.374 | 2.21 | 2.12 | 0.034 |
| Natal Males | 0.346 ± 0.389 | 1.41 | 0.89 | 0.373 |  | Immigration Event | 1.130 ± 0.422 | 3.09 | 2.67 | 0.008 |
|  |  |  |  |  |  |  |  |  |  |  |
| *External Takeover (N=34)* |  |  |  |  |  |  |  |  |  |  |
| Immigrant Males | -3.012 ± 0.809 | 0.05 | 3.72 | <0.001 |  |  |  |  |  |  |
| Natal Males | -2.021 ± 0.882 | 0.13 | 2.28 | 0.023 |  |  |  |  |  |  |
|  |  |  |  |  |  |  |  |  |  |  |
| *Abandonment (N=28)* |  |  |  |  |  |  |  |  |  |  |
| Partner Loss | 1.264 ± 0.499 | 3.54 | 2.54 | 0.011 |  |  |  |  |  |  |
| Unfamiliar Females | -2.500 ± 0.517 | 0.08 | 4.83 | <0.001 |  |  |  |  |  |  |
|  |  |  |  |  |  |  |  |  |  |  |


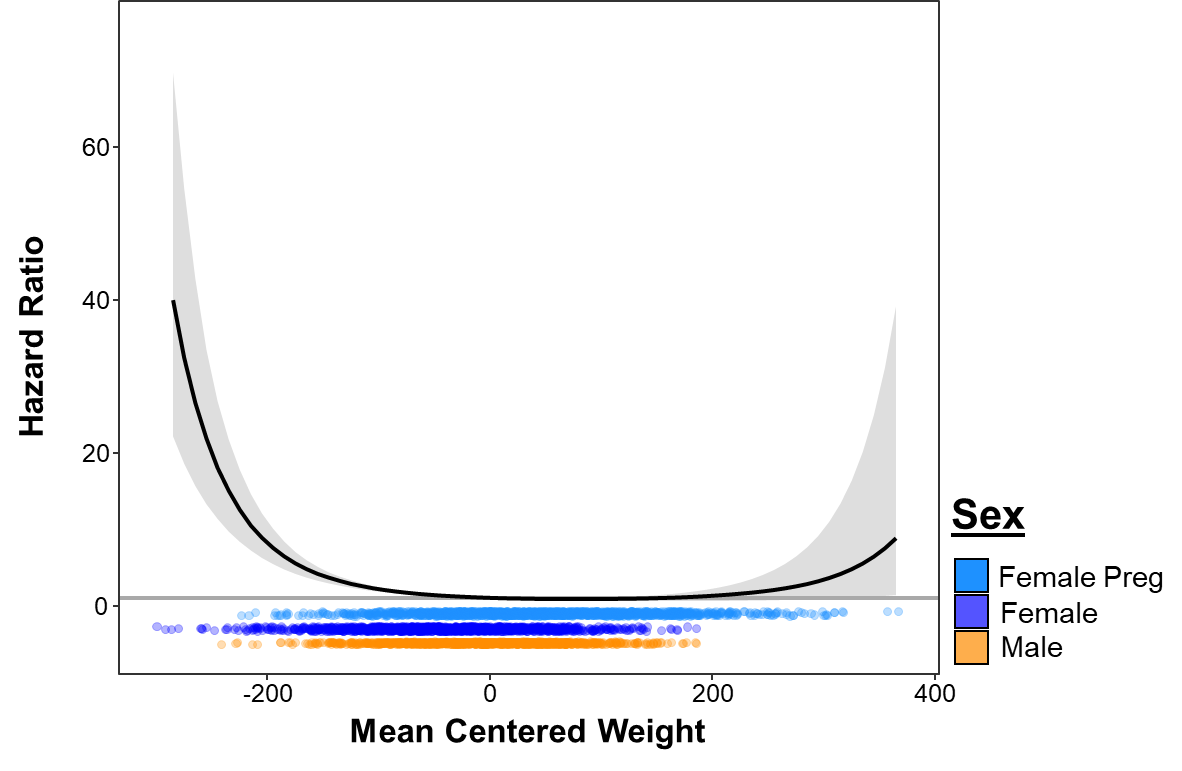


**SI 5:** Hazard plot of the effect of mean monthly morning body mass on the likelihood of mortality in dominant meerkats. Mean estimates (Solid Line) and accompanying 95% credible intervals (shaded ribbon) were predicted from a Cox proportional hazards model with both sexes modelled together. The weight distribution of males (orange) and females (blue) were plotted as jittered points along the x-axis, with female weights distinguished by whether the females were visibly pregnant (light blue) or not (dark blue), at any point during the month when a weight measurement was taken.

**SI 6: Impacts of subordinate immigrant males on dominant male tenure maintenance**

The competing risk model presented in this manuscript revealed that the presence of immigrant adult subordinate males had contrasting effects on the likelihood of males losing dominance to *internal displacement* and *external takeovers*. Predictions of the probability of maintaining dominance accounting for these contrasting effects indicated that the reduced risk of *external takeover* conferred by increasing numbers of subordinate immigrant males was enough to offset the cost they imposed on dominant males by increasing the risk of *internal displacement*, with immigrant subordinates only imposing a cost to the tenure maintenance of dominant males at unusually high numbers. To further test the robustness of this effect, we modelled all-cause dominance loss using a Cox proportional hazard model, essentially testing the effect of immigrant subordinate males on dominance loss irrespective of the cause of tenure loss. We controlled for the covariates included in our competing risks models and checked all terms did not violate the assumptions of proportional hazards.


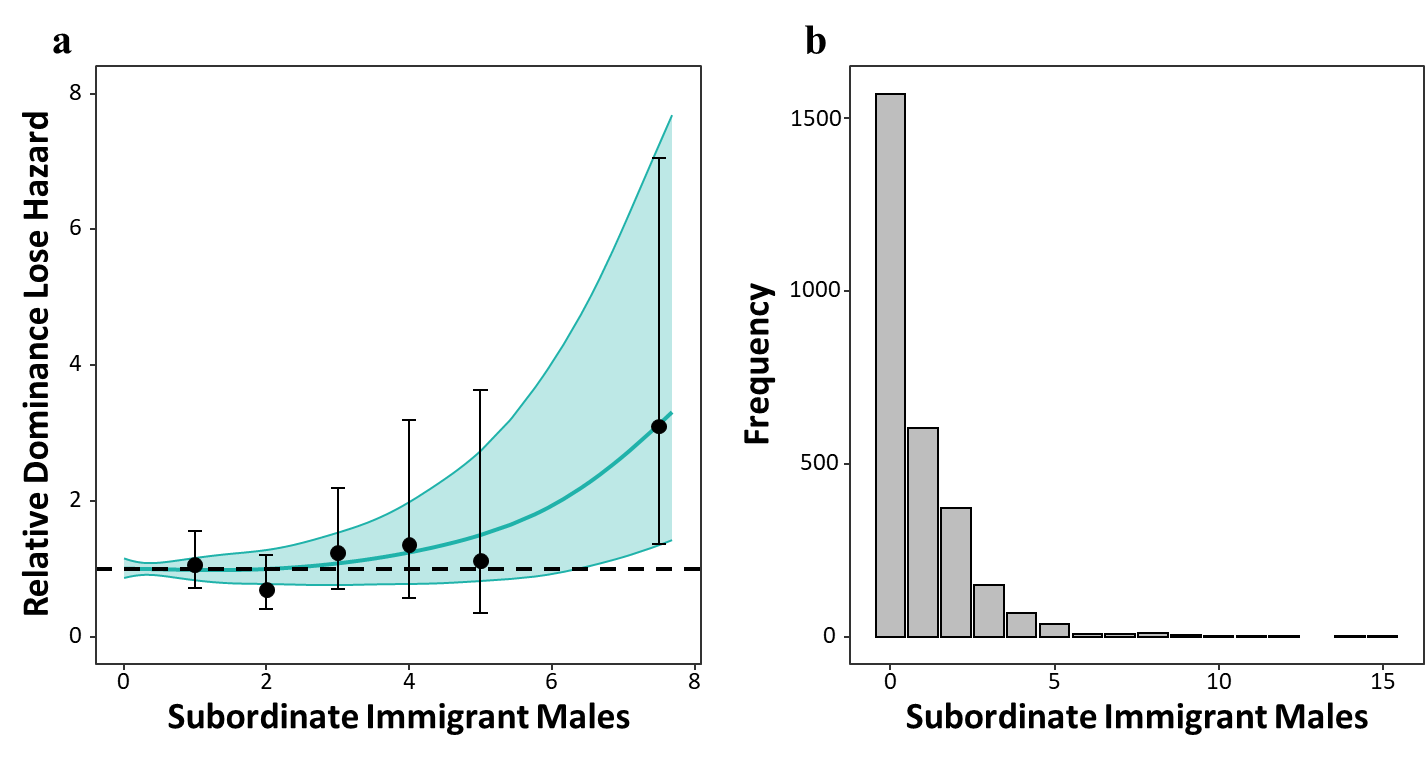


**Figure SI6.1:** The rate of dominance loss dependent on the number of adult subordinate immigrants resident in the group, predicted from models fitting subordinate immigrants as a categorical effect (mean effect black points and confidence intervals as error bars) and as a spline (mean effect green line and confidence intervals plotted as ribbon). All predicted rates are relative the rate of dominance loss when no subordinate immigrants are present. Categorical age levels were 1, 2, 3, 4, 5, & ≥ 6 years, with ≥ 6 years plotted at 7.5 on the x-axis.

When modelled as a linear fixed effect the number of immigrant subordinate males marginally increased the risk of dominant males losing their position. However, when this effect was further analysed by fitting immigrant subordinate males as a categorical effect or with a spline (Figure SI6a), it appeared this negative effect was driven by an increased risk of losing dominance associated with uncommonly high numbers of subordinate immigrants. At numbers below five, the presence of subordinate immigrant males had no tangible effect on the risk of losing dominance relative to when no subordinate immigrant males were present. This corroborates the predictions derived from our competing risks models that indicate that within the usual range of immigrant subordinate male numbers (Figure SI6b) they can offset their costs to the dominant male in terms of tenure maintenance.


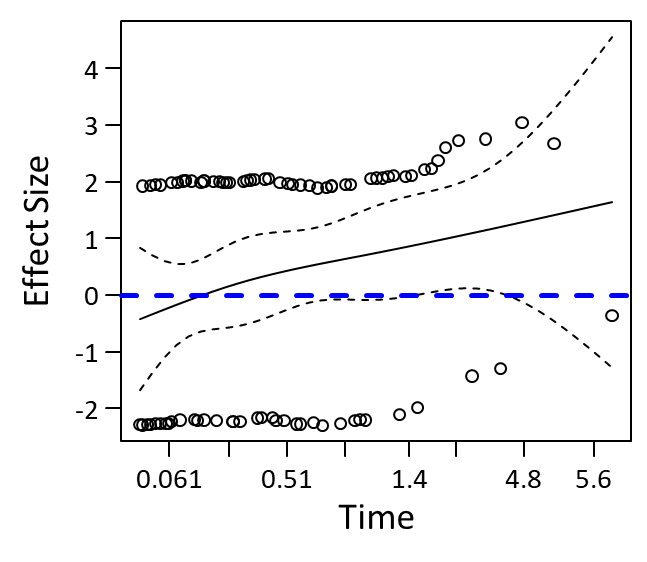


**SI7:** Proportional hazards diagnostic plot showing the change in the effect size of sex across the tenure on displacement with accompanying 95% confidence intervals. Females are the reference level, so this this suggests there is some evidence males may be more vulnerable to displacement later in tenure.


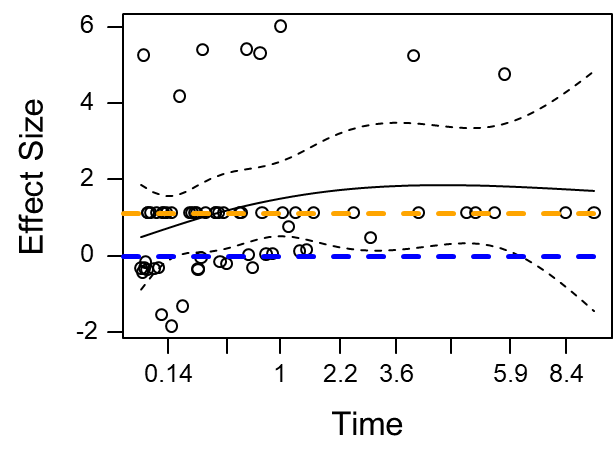


**SI 8:** Proportional hazards diagnostic plot showing the change in the effect size of male immigration across tenure on female displacement with accompanying 95% confidence intervals. This indicates the effect of Immigrant males is weaker at the very start of tenure before quickly stabilising, accounting for this effect with temporal interaction did not improve model fit and therefore was not included. The effect size reported in the model is also plotted (horizontal orange line)
